# Automated determination of peripheral nerve stimulation parameters to achieve desired effector response – a procedural routine, preliminary studies and proposal of improvements

**DOI:** 10.1186/1475-925X-12-11

**Published:** 2013-02-07

**Authors:** Paweł Maciejasz, Wiesław Marcol, Roman Paśniczek, Joanna Lewin-Kowalik, Klaus-Peter Hoffmann

**Affiliations:** 1 Institute of Metrology and Biomedical Engineering, Warsaw University of Technology, Warsaw, Poland; 2 DEMAR - LIRMM, University of Montpellier 2, INRIA, CNRS, Montpellier, France; 3 Department of Physiology, Silesian Medical University, Katowice, Poland; 4 Department of Rehabilitation, Medical University of Warsaw, Warsaw, Poland; 5 Department of Medical Engineering and Neuroprosthetics, Fraunhofer Institute for Biomedical Engineering IBMT, St. Ingbert, Germany

**Keywords:** Selective electric stimulation, Automated determination of stimulation parameters, Peripheral nerve, Multi-contact electrodes

## Abstract

**Background:**

The feasibility of selectively stimulating fascicles and fibers within peripheral nerves has been demonstrated by a number of groups. Although various multi-contact electrodes have been developed for this purpose, the lack of procedures for fast determination of stimulation parameters to produce the desired effector activity hampers the clinical application of these techniques.

In this paper, we propose an automated search routine that may facilitate the determination of stimulation parameters. To verify the routine's performance, we also developed an another routine that performs systematic stimulus–response mapping (the mapping routine).

**Method:**

The mapping routine performs systematic mapping of all possible combinations of the allowed stimulation parameters (i.e. combinations of electrode contacts used to provide the stimulus and sets of stimulus parameters values) and the observed displacements. The proposed automated search routine, similarly to the mapping routine, maps stimulation parameters to muscle responses, but it first investigates stimuli of the low charge and during the mapping process it compares the recorded responses with the desired one. Depending on the result of that comparison, it decides whether the use of a particular combination of electrode contacts should be further investigated or skipped.

Both approaches were implemented on a custom-made closed-loop FES platform and preliminary experiments were performed on a rat model. The rat's sciatic nerve was stimulated with a 12-contact cuff electrode and the resulting displacement of the rat's paw was determined using a MEMS accelerometer.

**Results:**

The automated search routine was faster than the mapping routine; however, it failed to find correct stimulation parameters in one out of three searches. This could be due to unexpectedly high variability in the responses to a constant stimulus.

**Conclusion:**

Our initial tests have proven that the proposed method determines the desired stimulation parameters much more quickly than systematic stimulus–response mapping. However, the factors influencing the variability of responses to constant stimuli should be identified, and their influence diminished; the remaining essential variability can then be identified. Thereafter, the criteria influencing the search process should be investigated and refined.

Further improvements to the search routine are also proposed.

## Background

State-of-the-art neuroprostheses are able to restore only a small part of body functions lost due to disease or injury [1]. To restore other functions such as vision or coordinated and accurate movement, only certain fibers or groups of fibers within the nerve need to be activated. A number of groups have proposed various multi-contact electrodes implanted around or within the nerve, which allow selective activation of particular nerve fibers through the choice of appropriate stimulation parameters (a review on this subject is available [2]). However, to take advantage of such interfaces, it is necessary to know which combination of electrode contacts (CEC) and which set of stimulus parameter values (SPV) should be used to activate particular nerve fibers in order to produce the desired response (i.e., the desired level of muscle or other effector activity). If electrodes with a high number of stimulation sites are used, there is a correspondingly large number of possible CEC and SPV combinations and manually checking all these combinations is tedious and time-consuming. For example, 10–15 hours were needed to perform an experiment during which the recruitment curves were determined for four muscles innervated by cat's sciatic nerve stimulated with a 12-polar cuff electrode [3].

Realistic computational models, like those described in [4,5], could be used to predict the CECs and SPVs required to achieve the desired response. However, prediction assumes the specific location of nerve fibers and fascicles within the nerve, which in fact varies among individuals, and knowledge of the exact positions of the electrode contacts around or within the nerve, which depends on the implantation procedure. Therefore, such models may and should be used to determine which CECs and SPVs should be investigated, which would decrease the number of combinations to be tested. However, they still cannot determine the exact combination of CECs and SPVs that would produce the desired response.

An alternative for manual determination of the desired stimulation parameters is an automated search. In the simplest case, the routine performs automated stimulus–response mapping by systematic verification of all the combinations of SPVs and CECs allowed by the operator. Such an approach reduces the search time since the automated setting of stimulation parameters is faster than manual setting but still generates the same number of pulses as manual mapping.

Automated routines, which adapt the parameters of the next stimuli to be investigated during the search based on the responses recorded for the already-generated stimuli, could further shorten the search time. However, such algorithms have been presented by few groups thus far [6,7].

Polasek et al. [6] proposed an adaptive binary routine automatically selecting stimulus amplitude or stimulus duration in order to determine muscle recruitment curves. The routine was applied to determine muscle recruitment curves when stimulating nerves of human arm [6] and leg [8,9] using a four-contact spiral nerve cuff electrode [6,8] and an eight-contact flat interface nerve electrode (FINE) [9].

Wilder et al. [7] proposed two routines for automated mapping of stimulation parameters with muscle responses from the nerve trunk stimulated by an electrode with multiple contacts. The first routine was used to determine the stimulation levels needed to produce perithreshold (i.e., small yet measurable) muscle activity. The second algorithm automatically determined the isometric force or EMG recruitment curves, based on the assumption that recruitment curves have a sigmoidal shape. Both routines were verified using a Utah Slanted Electrode Array with 100 stimulating contacts implanted in a cat's hind limb nerves [10].

However, the routines proposed by the both groups used only various SPVs for each of the electrode contacts separately and did not investigate the possibility of using a few electrode contacts concurrently. Furthermore, determining the recruitment curves for each CEC is not necessary if the desired response is already specified. In this case, investigation of the responses produced using particular CECs should be stopped once it becomes clear that these particular CECs do not lead to the desired response.

Certain controllers have been proposed to provide precise, time-varying effector response, i.e., muscle isometric force [11] and joint torque [12]; however, these controllers also require stimulus–response mapping during the initialization phase. Frankel [11] reported that minimizing the duration of the initialization processes is of critical importance to the use of such controllers in neuroprostheses.

Such a method is needed for the fast determination of stimulation parameters using multi-contact electrodes to produce the desired effector response. Therefore, we propose a routine that automatically searches for the combination of CECs and SPVs that will produce the desired effector response, hereafter referred to as “the automated search routine.”

In order to evaluate the performance of the automated search routine and compare it with the performance of routines systematically checking all combinations of SPVs and CECs allowed by the operator, we have also developed another routine that performs such systematic stimulus–response mapping (hereafter referred to as “the mapping routine”). Both routines were implemented on a custom-made closed-loop FES platform and preliminary experiments were performed on rat model.

## Methods

The manner of measuring the functional output of the stimulation also determines the way in which the desired response, i.e., the desired effect of the stimulation, is specified. The routines were developed and implemented to allow for specifying the desired response as either scalar (e.g., ENG or EMG signals, isometric force of isolated muscles) or vector (e.g., torque produced in the particular joint or displacement of a particular part of the body) values.

During the experiments, the desired response was defined as the two-dimensional displacement of rat's paw (i.e., the magnitude and direction of the maximal displacement from the initial position in the frontal plane) observed right after the stimulation. Therefore, we will hereafter use term “desired displacement” when referring to the desired response. However, other functional outputs, both scalar and vector, may be used by the routines in a similar way.

### The mapping routine

**Figure 1:**
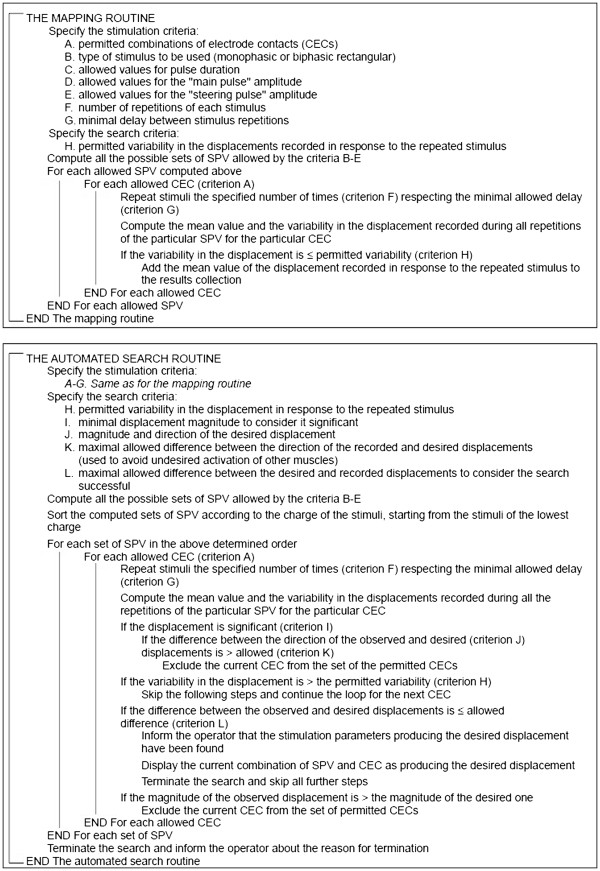
The pseudocode representation of the algorithms used by the two routines described in the paper.

This routine performs systematic mapping of all possible combinations of the allowed stimulation parameters (i.e., SPV and CEC combinations) and the observed displacements. It operates according to the algorithm presented in Figure 1.

To begin, the operator specifies the stimulation and search criteria, which are:

•Permitted combinations of electrode contacts (CECs): Checking all possible CECs usually is not needed, and the operator should therefore specify those that should be investigated. As the CECs that should be investigated will depend on the electrode geometry, the selection should be made based on the results of computational modeling or previous experience. For example, the operator may specify that only longitudinal or tri-polar configurations should be used for the cuff electrode.

•The operator defines each permitted CEC by specifying one of four possible states for each electrode contact in that configuration. These states are: cathode, anode, grounded, and not connected. The algorithm requires that for each CEC one cathode and at least one grounded contact, are selected to provide the “main pulse”. It is also possible to select another contact as an anode or a second cathode. That second contact would be used to provide the “steering pulse”. The “main pulse” is the one that is supposed to activate, i.e. to cause initiation of action potential propagation, in a group of nerve fibers within the nerve. The “steering pulse” is a pulse that is generated concurrently with the “main pulse” and has the same duration, but usually a lower charge as it is used for “steering” the excitation region within the nerve (this technique was first described by Sweeney [13]).

•Type of stimulus that should be used (e.g., monophasic or biphasic rectangular).

•The values allowed for pulse duration, “main pulse” amplitude, and “steering pulse” amplitude. “Steering pulse” amplitudes are defined as the fractions of the main pulse amplitude.

•Number of repetitions of each stimulus and minimal delay between the generation of two consecutive stimuli: The routine repeats each stimulus using the same CEC and SPV combination a specified number of times in order to determine the variability in the displacements for a particular stimulus. The typical number of repetitions of the same stimuli used by other authors is between three [6] and five [14] and the typical delay between consecutive stimuli is between 0.25 [6] and 2 s [14].

•Permitted variability in the displacements recorded in response to the repeated stimulus *F*: This allows skipping those combinations of CEC and SPV that produce unstable responses. It is computed using the following formula:

(1) $$F=\frac{\sum \sqrt{{\left(\bar{x}-x_{i}\right)}^{2}+{\left(\bar{y}-y_{i}\right)}^{2}}}{n}\frac{100}{\sqrt{{\bar{x}}^{2}+{\bar{y}}^{2}}}$$

where:

•n - Number of repetitions of the particular stimulus (i.e., the particular combination of SPV and CEC).

•x_i_, y_i_ - The horizontal (add-/abduction) and vertical (plantar-/dorsiflexion) components of the maximal displacement recorded during a particular repetition of the stimulus.

•$\bar{x},\bar{y}$ - Mean values of the horizontal (add-/abduction) and vertical (plantar-/dorsiflexion) components of the maximal displacements recorded during *n* repetitions of the stimulus.

Functionally, the variability *F* corresponds to the mean distance between each maximal displacement recorded during *n* repetitions of the same stimulus and the mean value of those maximal displacements. It is expressed as a percentage of the mean value of the maximal displacements recorded during *n* repetitions of this stimulus.

After initialization, the routine automatically determines all SPVs that satisfy the criteria specified by the operator and generates a series of stimuli for each combination of the determined SPVs and allowed CECs. The maximal value of the displacement produced by such stimulation is recorded and the mean value and the variability of the values recorded during *n* repetitions of a particular combination of SPV and CEC are calculated. If the variability is too high, the displacements recorded for that particular combination of SPV and CEC are not included in the results collection. At the end of mapping, all the results from the results collection are displayed and it is possible to select the combination of SPV and CEC that caused the paw's displacement closest to the desired one.

### The automated search routine

This routine automatically searches for the stimulation parameters producing the desired displacement of the rat's paw. During stimulation, it compares the recorded displacements with the desired one and, depending on the result of that comparison, decides whether the use of a particular CEC should be further investigated or skipped. Therefore, the search time may be significantly reduced. The routine operates according to the algorithm presented in Figure 1.

Before executing this routine, the operator should specify the same parameters as for the other routine, as well as:

•The magnitude and direction of the desired displacement.

•The maximal allowed difference between the desired and the recorded response that will be accepted, defined as the percentage of the magnitude of the desired displacement.

•The minimal magnitude of the paw's displacement to be considered as significant.

•The maximal allowed difference in the direction between the observed and the desired displacement, used to avoid undesired activation of other muscles.

After the parameters are specified, the routine determines all SPVs that satisfy the criteria specified by the operator and then sorts them according to the pulse charges. In the next step, pulses are generated starting from those of the lowest charge for each allowed CEC. However, if for a particular CEC the direction of the observed displacement is significantly different from that of the searched for displacement, or its magnitude is higher than that of the desired one, the CEC is excluded from the allowed CECs and will not be investigated for pulses with higher charge, since it could possibly cause even stronger undesired displacement. If the variability of the displacements recorded during the repetition of the particular SPV and CEC combination is higher than what is allowed, then the results are skipped. Last, if the variability in the displacements is not higher than the allowed value and the difference between the recorded displacement and the desired one is less than the accepted error, then the operator is informed that the stimulation parameters producing the specified displacement have been found and the search is terminated.

Alternatively, if all the SPVs have been tested or all CECs have been marked to be skipped, and the stimulation parameters producing the desired displacement have not been found, the search is terminated and the operator is informed about the reason for termination.

### The experimental set-up

The two proposed routines were implemented with customized software developed by our team using the Microsoft Visual Basic Express programming package. After verifying the correct operation of these routines during simulations and laboratory tests, the initial experiments in rats were performed.

For stimulation and signal acquisition, the programmable current stimulator with integrated signal analyzer PULSEGEN/ANA-16-10 (Creotech Ltd., Poland) was used. It has 16 current-controlled outputs and eight inputs for signal acquisition. Pulse duration and current amplitude on each output channel can be independently adjusted with a precision of up to 62 ns and 8 μA, respectively. The sampling rate of input channels is 60 kHz with a resolution of 8 bits. The custom software in which the proposed routines were implemented was installed on a laptop (Dell Studio 1537).

### The experimental procedure

The preliminary tests were conducted on one rat (race Wistar C). All procedures were approved by the Local Ethics Committee of Silesian Medical University. The duration of each experiment was limited to 2 hours because of the anesthesia.

**Figure 2:**
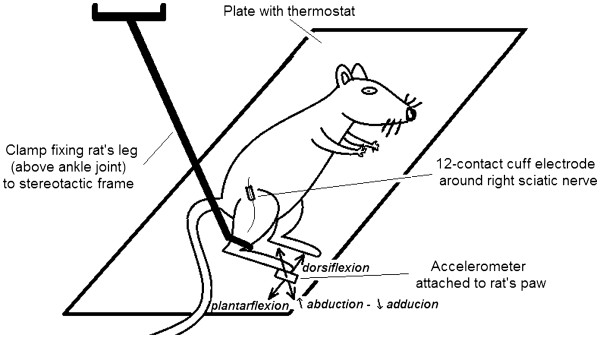
The schema of the set-up used during the experiments in rat.

**Figure 3:**
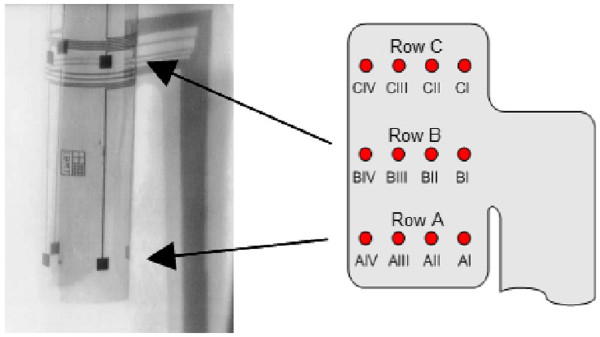
**A 12-contact cuff electrode.** A 12-contact cuff electrode the same as the one used in the experiments (left), and the symbols of the stimulating contacts used in the paper (right). Arrows indicate the location of rows A and B in the photograph. Row C is not visible.

The schema of the set-up used during that experiment is presented in Figure 2. The rat was anesthetized with chloral hydrate (0.42 g/kg body weight). A 12-contact cuff electrode (Fraunhofer IBMT, Germany; Figure 3) was implanted around the right sciatic nerve. A triaxial accelerometer (LIS3L06AL, STMicroelectronics, Switzerland) was attached to the right paw in order to calculate the paw displacement caused by the stimulation. The rat’s leg was attached to the stereotactic frame just above the ankle in a way that constrained movements of the leg above the ankle but did not restrict movements of the paw. Because the rat was anesthetized and the leg was not touching any object but the stereotactic frame, it was assumed that the paw returned to the same (initial) position after the movement caused by stimulation.

In order to evaluate the effect of the stimulation, the magnitude and direction of the maximal displacement of the rat's paw in the frontal plane was computed within the first 80 ms after stimulation by double integration of its acceleration. When performing the integration, the velocity and the acceleration of the paw at the start of stimulation was assumed to be equal to 0, since the paw was not moving at that moment.

The experiment consisted of three phases:

#### Phase 1 – stimulus–response mapping

At the beginning of the experiment, the stimulation parameters were systematically mapped to the rat's paw displacement using the mapping routine.

The permitted CECs were defined as follow (for symbols see Figure 3):

•One of the electrode contacts in row B was used to provide the “main pulse” (as a cathode).

•The corresponding contacts in rows A and C (i.e., AI and CI, if contact BI was used for stimulation) were grounded.

•In addition, one of the contacts in row B, other than the one used to provide the “main pulse,” could be used to provide the “steering pulse” (either cathodic or anodic) or be grounded.

In total, 40 CECs satisfying the above criteria were defined.

The following search criteria were used:

•Type of stimulus: monophasic rectangular.

•Permitted stimulus durations: 10, 20 and 40 μs. The short pulse widths were chosen to increase the threshold differences between nerve fibers of different diameters [15].

•Permitted “main pulse” amplitudes: 50, 100, 200, 400 μA.

•Permitted “steering pulse” amplitudes: 50 and 100% of the “main pulse.”

•Number of repetitions of each stimulus: 3.

•Minimal delay between stimulus repetitions: 400 ms.

•Allowed variability *F* in displacements for the same stimulus: 10% of the mean value of maximal displacements recorded during three repetitions of the same stimulus (see eq. 1).

#### Phase 2 – automated determination of stimulation parameters

After the systematic stimulus–response mapping, three displacements of the rat's paw recorded during this mapping were selected and used as the desired displacements for the search performed with the automated search routine.

The automated search routine was performed three times, once for each of the selected displacements. The search criteria and the permitted CECs for these searches were the same as during the execution of the mapping routine, in order to be able to compare the results obtained during execution of both routines. In addition, the following search criteria were specified:

•Maximal allowed difference between the recorded and the desired displacements to consider the search successful: 15% of the magnitude of the desired displacement.

•Minimum magnitude of the displacement to consider it as significant: 0.5 mm

•Maximal allowed difference between the direction of the recorded and the desired displacements (used to avoid undesired activation of other muscles): 60°.

There are not much data available about the variability of responses that could be expected, therefore the values of the above criteria were selected based on the responses observed during initial tests of the experimental set-up [16].

#### Phase 3 – verification of the determined stimulation parameters

At the end of the experiment, stimulation was performed once again using the combination of SPV and CEC determined by the automated search routine for one of the searched for displacements, in order to verify whether the determined stimulation parameters allowed it to achieve the desired displacement. The stimulus was repeated three times and the mean value and variability of the recorded displacements were calculated.

## Results

### Phase 1 – stimulus–response mapping

**Figure 4:**
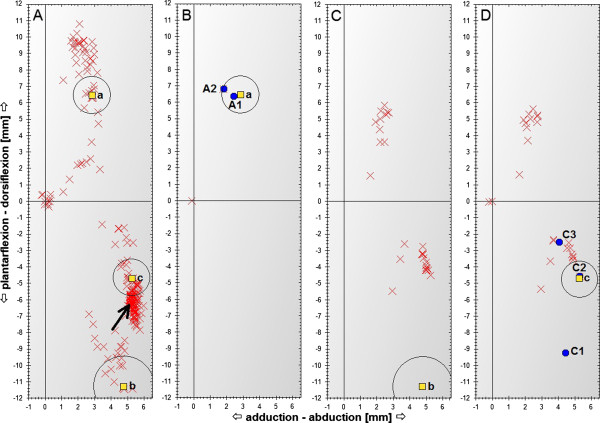
**Maximal displacements of the rat's paw recorded during the performed experiments.** Maximal displacements of the rat's paw recorded during searches with the mapping routine **(A)** and the automated search routine for three different desired responses **(B-D)**. Each X sign corresponds to the mean displacement of the rat's paw during repetition of the particular stimuli three times. For the explanation of other symbols, see Table 2.

During the mapping of stimulation parameters with paw displacements, 768 various stimuli - i.e., various CEC and SPV combinations - were generated, with each stimuli repeated three times. The search duration was 27 min 15 s (see Table 1). The rat's paw displacements recorded during the execution of the mapping routine are presented in Figure 4A and marked with *X*. Both plantarflexion and dorsiflexion displacements with small abduction of the paw can be observed. There is also a high concentration of displacements corresponding to plantarflexion combined with abduction of the paw (area indicated by an arrow in Figure 4A). Most of these displacements were observed for stimuli with a charge higher than 10 nC. Because displacements in other directions were mostly recorded for stimuli with lower charges, we believe that displacements in the indicated area were observed mostly when all or almost all fibers within the nerve were activated and thus all muscles innervated by the sciatic nerve were contracting in the same time.

**Table 1:** The execution durations and effective stimulation frequencies during particular searches

**Operation**	**Number of various stimuli (i.e., SPV and CEC combinations) tested**	**Number of significant displacements (i.e., mean magnitude > 0.5 mm)**	**Execution duration**	**Effective stimulation frequency [Hz]**
Phase 1: Mapping of stimulation parameters with muscle responses (Figure 4A)	768	362	27 min 15 s	1.41
Phase 2: Search for stimulation parameters producing response *a* (Figure 4B)	133	4	4 min 24 s	1.51
Phase 2: Search for stimulation parameters producing response *b* (Figure 4C)	265	58	8 min 54 s	1.49
Phase 2: Search for stimulation parameters producing response *c* (Figure 4D)	259	52	9 min 38 s	1.34

Each stimulus was repeated three times for each combination of allowed SPVs and CECs.

### Phase 2 – automated determination of stimulation parameters

From the mapping results in phase 1, three significantly different displacements were selected. They are marked in Figure 4A with squares and *a*, *b* and *c*. When choosing these displacements, attention was paid to select displacements:

•In different directions – to ensure that these displacements were caused by the activation of different muscles.

•In directions for which a significant number of displacements was observed during stimulus–response mapping – to avoid using accidental displacements.

•With both maximal and intermittent magnitude values, as compared with other displacements observed during stimulus–response mapping – to verify whether the routine was able to find stimulation parameters corresponding to various muscle activation levels.

Those displacements were used as the desired ones for three searches performed using the automated search routine. The displacements recorded during these searches are presented (*X* signs) in Figure 4B, C and D for desired displacements *a*, *b* and *c*, respectively. For comparison, the searched for displacements are presented in these figures and marked with squares and the appropriate letter (*a*, *b* or *c*). The circles around the desired displacements in Figure 4 indicate the maximal allowed difference between the recorded and desired displacements to consider the search successful, which was set to 15% of the magnitude of the desired displacement. Therefore, the absolute value of the maximal allowed difference was higher for the desired displacements having higher absolute magnitude (e.g. displacement *b*).

The routine found the stimulation parameters producing displacements *a* and *c*. The found displacements are marked in Figure 4B and D with blue circles and the symbols *A2* and C*2* for the desired displacements *a* and *c*, respectively. The stimulation parameters producing displacement *b* were not found. The search was terminated when all allowed SPVs had been generated for all unskipped CECs.

**Figure 5:**
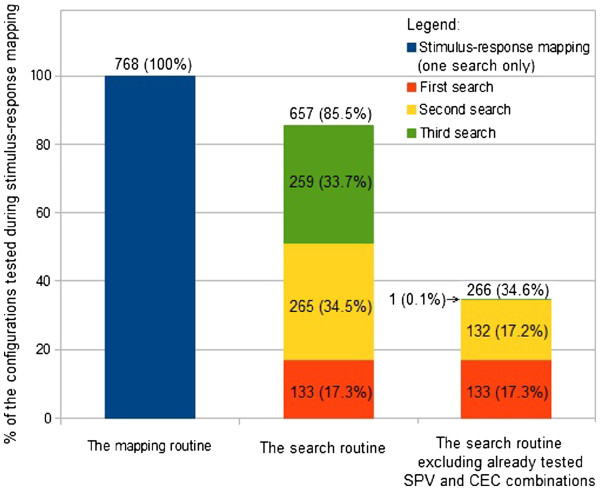
**Comparison of the number of various SPV and CEC combinations tested during the performed experiment.** Comparison of the number of various SPV and CEC combinations tested during execution of the mapping routine and three executions of the automated search routine. Numbers above bars indicate the total number of configurations tested during all the executions of the particular routine. Values in parentheses provide a percentage comparison of the configurations tested during particular searches with the number of configurations tested during stimulus–response mapping.

The search time and number of tested SPV and CEC combinations for each search are presented in Table 1. The use of the proposed automated search routine allowed us to reduce the search time and the number of generated stimuli compared with the systematic stimulus–response mapping routine. During execution of the mapping routine, 768 SPV and CEC combinations were tested and it took more than 27 minutes, whereas during each execution of the automated search routine no more than 270 stimuli were tested and the routine execution duration was less than 10 minutes each time (see Figure 5 and Table 1 for comparison).

During the search for stimulation parameters producing displacement *a*, the first significant response with the allowed variability that was observed had already satisfied the search criteria (see displacement *A2* in Figure 4B). However, before the generation of the stimulus that produced the displacement, 132 other stimuli with lower charge were also generated, with only three of them producing significant displacement but with too high variability.

### Phase 3 – verification of the determined stimulation parameters

At the end of the experiment, to ascertain whether the combination of SPV and CEC found by the automated search routine had achieved the desired displacement, the stimulation parameters found by the automated routine to produce displacement *c* (i.e., the combination of SPV and CEC that produced displacement *C2*) were used to generate three consecutive stimuli. The mean paw displacement observed during this stimulation was calculated and is marked as *C3* in Figure 4D. There was a significant difference between displacements *C2* and *C3*, despite the use of the same stimulation parameters.

When a search using the systematic stimulus–response mapping routine was performed, all combinations of permitted SPVs and CECs were tested. The same SPVs and CECs were permitted when performing a search using the two routines; therefore, it was possible to compare the displacements recorded for the same combination of SPV and CEC during the execution of both routines. When displacements *A2* and *C2* recorded during execution of the automated search routine were compared with displacements recorded for the same combinations of SPV and CEC generated during execution of the mapping routine (marked in Figure 4B and D with a blue circle and symbols *A1* and *C1,* respectively), a significant difference between *C1* and *C2* was observed (see Figure 4D) and there was a much smaller difference between *A1* and *A2* (see Figure 4B). The stimulation parameters and displacements recorded for the stimuli described above are compiled in Table 2.

**Table 2:** **Stimulation parameters and recorded responses for stimuli marked in Figure**4

**ID**	**Stimulation parameters**	**Stimulus charge [nC]**	**Paw displacement**		
			**Magnitude [mm]**	**Direction [º]**	**Variability [% of magnitude]**
*a*	40 μs, BI: -400 μA, BII: -200 μA, AI + CI	24	7.05	66.2	2.0
*A1*	10 μs, BII: -200 μA, none, AII + CII	2	6.81	68.9	17.4
*A2*	Same as stimulus A1	2	7.02	74.9	8.5
*b*	20 μs, BIII: -400 μA, BIV: 200 μA, AIII + CIII	12	12.27	−67.1	4.2
*c*	40 μs, BI: -200 μA, BII: -100 μA, AI + CI	12	7.07	−41.8	4.7
*C1*	40 μs, BIII: -400 μA, none, AIII + BIV + CIII	16	10.25	−64.3	0.6
*C2*	Same as stimulus C1	16	6.99	−40.5	3.1
*C3*	Same as stimulus C1	16	4.77	−31.6	4.4

Responses *a*, *b* and *c* were chosen from the results of the stimulus–response mapping (phase 1 of the experiment) and used as the desired responses for the search performed using the automated search routine. *A2* and C*2* are responses produced using stimulation parameters determined in phase 2 of the experiment by the automated search routine for responses *a* and *c*, respectively. *A1* and C*1* are the responses recorded for the stimuli with the same parameters as *A2* and C*2*, but generated during phase 1 of the experiment. Response C*3* was recorded at the end of the experiment when verification using the same stimulation parameters as for C*2* was performed (phase 3 of the experiment). Mean response values are provided (each stimulus was repeated three times every 400 μs). The stimulation parameters are provided in the following order: stimulus duration, electrode contact providing the “main pulse” and its amplitude, electrode contact providing the “steering pulse” and its amplitude, electrode contacts being grounded. For an explanation of the symbols for the electrode contacts, see Figure 3.

The combination of CEC and SPV that produced displacements *C* (i.e., *C1*, *C2* and *C3*) was used for stimulation nine times during the whole experiment – three times during stimulus–response mapping, three times when performing a search for stimulation parameters producing displacement *c*, and three times when the parameters determined by the proposed routine were verified. In each case, the variability in the displacements observed when repeating this stimulus three times during particular phases of the experiment was less than 5% of the mean magnitude of the recorded displacements (see Table 2). During stimulus–response mapping (displacement *C1*), this variability was even less than 1%; however, the difference between the mean values of *C1* and *C2* was 69% of the mean magnitude of all nine *C* displacements and the difference between *C2* and *C3* was 32% of the same mean magnitude (see Figure 4C). Thus, it appears that the delay between executions of these stimuli can have an influence on the differences between the recorded displacements. During each part of the experiment, the same stimuli were repeated every 400 ms, whereas the delay between generations of the same stimuli during various parts of the experiment was much higher. The delay between the stimuli which produced *C1* and *C2* was approximately 55 minutes and the one between *C2* and C*3* was 3 minutes and 20 seconds.

## Discussion

The main goal of the proposed routine is to allow fast and reliable determination of the stimulation parameters needed to achieve the specified response of muscles or other effectors. The initial tests, the results of which we present in this paper, show that the routine allowed a rapid determination of stimulation parameters producing the desired responses (see Table 1) but failed to determine the right stimulation parameters in one out of three cases. This occurred because of the high variability in the responses, which caused skipping of the correct stimulation parameters, namely the combination of SPV and CEC that produced displacement *b* during phase 1 of the experiment.

The significant variance of the displacements for the same stimuli during various parts of the experiment indicates that the use of this routine is questionable, since the stimulation parameters that are valid at the moment of determination are not necessarily capable of achieving reproducible results when repeated. However, the same problem would arise for any other routine proposed for the same purpose. It is thus necessary to determine the factors that cause the high variation in responses to a constant stimulus, diminish the influence of these factors, and determine the remaining essential variability. Only once this is accomplished can the routine proposed by our group, or any other routine proposed for the same purpose, undergo validation procedures that take into account the remaining variability. The factors that could cause such a high variability will be discussed below.

Although, the results presented in this paper do not allow to validate the method, they allow us to make propose some improvements that may be introduced to the algorithm. Those improvements will be described at the end of the discussion section. We believe that improving the experimental conditions in order to reduce the variability of the observed responses, together with the implementation of the proposed modifications, will greatly increase the performance of the proposed routine and allow for its application for fast determination of the stimulation parameters.

### The variability in the responses to the constant stimulus

In essence, the factors that could cause variability in the responses to the constant stimulus may be divided into three categories:

1. Factors related to the change in stimulation conditions (due to stimulator drift, electrode displacement, nerve drying, or nerve swelling).

2. Factors related to a change in the excitability of the nerve and muscle cells (due to fatigue, potentiation phenomena, or a change in tissue temperature).

3. Factors related to the measurement method.

Although most of these factors should have had only a minor influence during our experiment, it is nevertheless worthwhile to discuss them in greater detail.

#### Factors related to the change in stimulation conditions

Due to technical limitations, the stimuli generated by an electrical stimulator for the same input parameters may vary within a limited range. However, upon testing the stimulator used in the experiment, the variability in the amplitude of the rectangular pulses generated for the constant input parameters was less than 2% and no variability in pulse duration was observed. Therefore, it can be inferred that the influence of stimulator drift on the variability in the observed displacements wasn't significant in our experiment.

Another possibility is that even though the stimulation parameters are held constant, other stimulation conditions may change and thus influence the threshold values for the activation of particular fibers, thereby causing a change in the responses observed for the same stimulus. Such change can be brought about by factors such as nerve drying or swelling or electrode movement. These factors play an especially significant role in acute experiments such as ours, although in our previous work [16] electrodes were implanted five days before the experiment and when they were explanted, a stable and very good contact between electrode and nerve was observed. Nevertheless, considerable variability in the displacement responses to the constant stimulus was observed, as in our current experiment, which suggests that factors related to stimulation conditions were not the main factors causing the variability of responses.

#### Factors related to the change in nerve and muscle cell excitability

Other factors like anesthesia, temperature and stimulation frequency may also influence the excitability of the stimulated nerve and muscles, which could explain the variability in muscle response to the same stimulus. The anesthesia used in this experiment (chloral hydrate) is regularly used in animal research, but we are not aware of any published study showing its influence on nerve excitability over time. A thermostat was used to ensure the constant temperature of the rat's body during the experiment, so changes in body temperature are also unlikely to explain our results.According to Wilder [7], a stimulation frequency equal to or higher than 2 Hz may induce muscle fatigue. During our experiment, the minimum delay between two consecutive stimuli was set to 400 ms, which is equal to 2.5 Hz. However, due to the time-consuming transmission, processing and real-time visualization of the recorded signals, the effective stimulation frequency during execution of the search routine was about 1.5 Hz (see Table 1). Therefore, muscle fatigue was unlikely to have occurred. On the other hand, repetitive stimulation, even at low frequency, can increase the force of a muscle contraction compared with a single-pulse stimulation, which is known as the “staircase potentiation” phenomenon. As shown by Krarup [17], this potentiation can be observed during repetitive generation of supra-maximal stimuli with a frequency as low as 2 Hz. Further studies are thus needed to determine the maximal stimulation frequencies at which the search can be performed without a potentiation effect being observed.

#### Factors related to the measurement method

Even for the same responses, the result of the measurement may differ if the measurement method is not sufficiently reliable. In this experiment, the MEMS accelerometer was used to measure paw displacement because it is easy to apply in practical scenarios. However, the signals it produces are not always reliable, and the double integration of these signals in order to calculate displacement may lead to significant errors [18]. In order to determine the precision of the displacement using our method, we have performed characterization of our experimental set-up. The results showed that the measurements were not precise (the mean error was 23.8% of the measured displacement), but repeatable (the mean standard deviation of the repeated measurements was 5.3% of the computed displacement).

Thus, the measurement method chosen for this experiment may have been an important factor of the variability in the observed displacements. A more reliable method is thus needed to measure effector response. However, for the binary search routine developed by Polasek, modifications in the routine were necessary to avoid getting stuck when the response to a stimulus with a low value was higher than the response to a stimulus with a high value, due to the variability in response amplitude (for more details see Appendix II to [6]). This variability was observed, even though EMG signals were used as input for the routine, the measurement of which is more reliable than the measurement of displacement using our approach. Thus, application of a reliable measurement method does not guarantee that stable responses will be obtained.

### Decreasing search duration

One of the objectives of the proposed method was to determine the parameters of stimulation relatively quickly. It is not possible to compare the proposed method with the other existing, because so far no equivalent method has been proposed. It is not possible to compare these methods with those proposed by Polasek [6] and Wilder [7], because their methods used only various SPVs for each of the electrode contacts separately and did not investigate the possibility of using a few electrode contacts concurrently. Also in most of the publications in which stimulus–response mapping of multi-contact electrodes was performed, no information was given on whether the stimulation parameters were set automatically or manually, the duration of the search procedure, or the number of the various pulses that were generated during the search [13,19,20]. Therefore, we decided to compare performance of the proposed automatic search routine versus performance of the automatic stimulus–response mapping routine that we have also implemented and tested for the same permitted SPVs and CECs. The results we obtained (Figure 5) show that the automated search-routine reduced search duration by at least 66% percent for a single search as compared to systematic stimulus–response mapping. However, the number of the tests performed is too low to allow definitive conclusions.

### Decreasing pulse charge

The other aim of the proposed method was to decrease the charge of the pulse needed to achieve the desired displacement. This would help to avoid nerve damage and decrease the power demands for prolonged stimulation. Therefore, the proposed routine first produces stimuli with the lowest charges for all allowed CECs and then increases the charge in a stepwise fashion. Hence, if the routine succeeds, we can obtain the parameters of the stimulus with the lowest charge to produce the response. The use of this routine resulted in a significant reduction in the charge for the stimulus producing displacement *a* (see Table 2: 24 nC during stimulus–response mapping and only 2 nC for the stimulus producing displacement *A2*, for which the parameters were found using the proposed routine). However, the charge of stimulus *C* found using the automated search routine during phase 2 of the experiment was higher than the charge of stimulus *c* during stimulus–response mapping (see Table 2). This was rather unexpected since the stimulus with the same parameters as the one that produced displacement *c* during execution of the mapping routine should have been tested by the automated search routine before testing stimuli with higher charges. However, the CEC that produced displacement *c* during the execution of the mapping routine produced a significantly different displacement for a pulse with a small charge during execution of the automated search routine and was therefore skipped during the further search.

### Improving search criteria

Skipping the CEC that could have produced the desired response when used with a SPV of higher charge might have been caused by the high variability in the displacements for the same stimulus. However, it may also have been due to overly strict criteria for CEC exclusion. Therefore, the CEC exclusion criteria should be investigated and refined to avoid exclusion of CECs producing the desired responses. When choosing the search criteria, there is always some trade-off between search time, number of unintended activations of other effectors, and the certainty to finding the right stimulation parameters to produce the desired response. Nevertheless, there is room for improvement. We observed that it was mainly the displacements with the lowest amplitudes that had significant variation Therefore, choosing a higher displacement magnitude to be regarded as significant should be considered.

### Further improvements to the proposed routine

After this initial experiment, proposals to improve the routine can be made:

1. The automated search routine significantly reduced the execution time in comparison with systematic stimulus–response mapping. However, if the routine is executed many times in a row in order to find stimulation parameters producing various responses, the same stimuli may be repeated during each search. This was the case for responses *b* and *c*, because the direction of the desired displacement in both cases did not differ by much (as can be seen from the comparison of desired and recorded responses in Figure 4C and D). Therefore, before generating a stimulus, it should be determined whether this stimulus was generated during recently performed searches. If this is the case, the stimulus should not be generated and the previously recorded response for the particular SPV and CEC combination should be used instead. As may be seen in Figure 5, this would have reduced the total number of configurations tested during the three executions of the automated search routine from 657 to 266.

2. During each search, a high number of stimuli was generated before any significant response was observed. This number would have been even higher if a lower rate had been selected for the increase in stimulus parameters, because stimulus amplitude and duration are increased in a stepwise manner starting from the lowest permitted values and using a constant ratio, irrespective of whether any significant response is observed. Using two different rates of increase, which would be considerably higher if the last generated stimulus did not produce a significant response and lower if the response was significant, would reduce the number of generated stimuli that produce insignificant responses and thus speed up the search process.

3. The proposed routine skips the CEC if the recorded response has a magnitude higher than the desired one, and this occurs irrespective of the direction of the recorded response. In the case when the rates of increase in the stimulus parameters are too high, it may happen that, in a series of two consecutive stimuli using the same CEC, the charge of the first one is too low while the charge of the second is too high to produce the desired response. As a consequence, this CEC will be skipped and the stimulation parameters producing the desired response may not be found. Therefore, when the response magnitude is too high, but the direction of the recorded response is similar to the direction of the desired one, the next stimulus using the same CEC should be generated, but with a charge value in between the values of the two previous stimuli.

4. The use of the steering current does not always modify the direction of the observed displacement, but it increases the number of configurations to be tested and the pulse charge . On the other hand, in some cases an additional grounded contact can be used to modify the direction of the displacement . Therefore, a method should be built into the algorithm for fast verification if a particular CEC allows the achievement of better responses than those obtained using similar CECs. Such a method could, for example, incorporate the determination of overlap between effectors activated by various CECs.

## Conclusions

We have proposed a method for the automated determination of stimulation parameters to produce a desired effector response. This type of method, in combination with multi-contact electrodes, may broaden the range of application for selective stimulation techniques in medicine. Our initial tests have proven that the proposed method determines the desired stimulation parameters much more quickly than systematic stimulus–response mapping. However, the variability in the responses to the same stimulus generated in various parts of the experiment was quite high. The factors influencing this variability should be identified, and their influence diminished; the remaining essential variability can then be identified. In particular, a more reliable measurement method should be considered. Only once this is accomplished can the routine proposed by our group, or any other routine proposed for the same purpose, undergo validation procedures. Thereafter, the criteria influencing the search process should be investigated and refined. We have also proposed a number of modifications that may further improve the performance of the search routine.

## Competing interests

The authors declare that they have no competing interests.

## Authors’ contributions

PM, RP and KPH contributed to the development of the stimulation strategies. PM, WM, JLK contributed to the verification of the developed strategies during animal experiments. All the authors contributed to the structure of the paper, read and approved the manuscript.

## References

[B1] Hoffmann KP Dehm J VDE-Studie zum Anwendungsfeld Neuroprothetik 2005 Frankfurt: VDE [publication in German]

[B2] Yoshida K Farina D Akay M Jensen W Multichannel intraneural and intramuscular techniques for multiunit recording and use in active prostheses Proc IEEE 2010 98 432 449

[B3] Grill W Veraart C Mortimer J Nagel JH, Smith WM Selective activation of peripheral nerve fascicles: use of field steering currents Annual International Conference of the IEEE Engineering in Medicine and Biology Society: 31 0ctober - 3 November, 1991, Orlando 1991 Piscataway: IEEE Service Center; Part 2 of 5 904 905

[B4] Raspopovic S Capogrosso M Micera S A computational model for the stimulation of rat sciatic nerve using a transverse intrafascicular multichannel electrode IEEE Trans Neural Syst Rehabil Eng 2011 19 333 344 21693427 10.1109/TNSRE.2011.2151878

[B5] Schiefer MA Triolo RJ Tyler DJ A model of selective activation of the femoral nerve with a flat interface nerve electrode for a lower extremity neuroprosthesis IEEE Trans Neural Syst Rehabil Eng 2008 16 195 204 18403289 10.1109/TNSRE.2008.918425 PMC2920206

[B6] Polasek KH Clinical implementation of nerve cuff electrodes for an upper extremity neuroprosthesis PhD Thesis 2007 Cleveland, Ohio: Case Western Reserve University, Department of Biomedical Engineering

[B7] Wilder AM Hiatt SD Dowden BR Brown NAT Normann RA Clark GA Automated stimulus–response mapping of high-electrode-count neural implants IEEE Trans Neural Syst Rehabil Eng 2009 17 504 511 19666339 10.1109/TNSRE.2009.2029494

[B8] Polasek KH Hoyen HA Keith MW Kirsch RF Tyler DJ Stimulation stability and selectivity of chronically implanted multicontact nerve cuff electrodes in the human upper extremity IEEE Trans Neural Syst Rehabil Eng 2009 17 428 437 19775987 10.1109/TNSRE.2009.2032603 PMC2927980

[B9] Schiefer MA Polasek KH Triolo RJ Pinault GCJ Tyler DJ Selective stimulation of the human femoral nerve with a flat interface nerve electrode J Neural Eng 2010 7 26006 9?pp 10.1088/1741-2560/7/2/026006 20208125 PMC2915830

[B10] Dowden BR Wilder AM Hiatt SD Normann RA Brown NAT Clark GA Selective and graded recruitment of cat hamstring muscles with intrafascicular stimulation IEEE Trans Neural Syst Rehabil Eng 2009 17 545 552 19696002 10.1109/TNSRE.2008.2011988

[B11] Frankel MA Dowden BR Mathews VJ Normann RA Clark GA Meek SG Multiple-input single-output closed-loop isometric force control using asynchronous intrafascicular multi-electrode stimulation IEEE Trans Neural Syst Rehabil Eng 2011 19 325 332 21385670 10.1109/TNSRE.2011.2123920

[B12] Qi H Tyler DJ Durand DM Neurofuzzy adaptive controlling of selective stimulation for FES: a case study IEEE Trans Rehabil Eng 1999 7 183 192 10.1109/86.769409 10391589

[B13] Sweeney JD Ksienski DA Mortimer JT A nerve cuff technique for selective excitation of peripheral nerve trunk regions IEEE Trans Biomed Eng 1990 37 706 715 10.1109/10.55681 2394459

[B14] Tarler MD Mortimer JT Linear summation of torque produced by selective activation of two motor fascicles IEEE Trans Neural Syst Rehabil Eng 2007 15 104 110 17436882 10.1109/TNSRE.2007.891377

[B15] Gorman PH Mortimer JT The effect of stimulus parameters on the recruitment characteristics of direct nerve stimulation IEEE Trans Biomed Eng 1983 30 407 414 6604691 10.1109/tbme.1983.325041

[B16] Maciejasz P Marcol W Paśniczek R Dörge T Mandl T, Martinek J, Bijak M, Lanmüller H, Mayr W, Pichler M An experimental setup for stimulation selectivity measurement – variability of the muscle responses to the constant stimulus Proceedings of the 10th Vienna International Workshop on FES and the 15th IFESS Annual Conference: 8–12 September 2010, Vienna 2010 Vienna: Medical University of Vienna & Austrian Society for Biomedical Engineering (ÖGBMT) & International FES Society (IFESS) 142 144

[B17] Krarup C Enhancement and diminution of mechanical tension evoked by staircase and by tetanus in rat muscle J Physiol 1981 311 355 372 7264972 10.1113/jphysiol.1981.sp013589 PMC1275414

[B18] Woodman OJ An introduction to inertial navigation Technical Report 696 2007 Cambridge: University of Cambridge

[B19] Badia J Boretius T Andreu D Azevedo-Coste C Stieglitz T Navarro X Comparative analysis of transverse intrafascicular multichannel, longitudinal intrafascicular and multipolar cuff electrodes for the selective stimulation of nerve fascicles J Neural Eng 2011 8 036023 13?pp 10.1088/1741-2560/8/3/036023 21558601

[B20] Tarler MD Mortimer JT Selective and independent activation of four motor fascicles using a four contact nerve-cuff electrode IEEE Trans Neural Syst Rehabil Eng 2004 12 251 257 10.1109/TNSRE.2004.828415 15218938

